# The Mechanism of Poly-Galloyl-Glucoses Preventing Influenza A Virus Entry into Host Cells

**DOI:** 10.1371/journal.pone.0094392

**Published:** 2014-04-09

**Authors:** Hu Ge, Ge Liu, Yang-Fei Xiang, Yu Wang, Chao-Wan Guo, Nan-Hao Chen, Ying-Jun Zhang, Yi-Fei Wang, Kaio Kitazato, Jun Xu

**Affiliations:** 1 School of Pharmaceutical Sciences & Institute of Human Virology, Sun Yat-Sen University, Guangzhou, China; 2 Division of Molecular Pharmacology of Infectious agents, Department of Molecular Microbiology and Immunology, Graduate School of Biomedical Sciences, Nagasaki University, Nagasaki, Japan; 3 Division of Biomedicine Research and Development Center, Guangdong Provincial Key Laboratory of Bioengineering Medicine, National Engineering Research Center of Genetic Medicine, Jinan University, Guangzhou, China; 4 College of Pharmacy, Jinan University, Guangzhou, China; 5 Kunming Institute of Botany, the Chinese Academy of Sciences, Kunming, China; German Primate Center, Germany

## Abstract

Hemagglutinin (HA) is essential for Influenza A virus infection, but its diversity of subtypes presents an obstacle to developing broad-spectrum HA inhibitors. In this study, we investigated the molecular mechanisms by which poly-galloyl glucose (pGG) analogs inhibit influenza hemagglutinin (HA) *in vitro* and *in silico*. We found that (1) star-shaped pGG analogs exhibit HA-inhibition activity by interacting with the conserved structural elements of the receptor binding domain (RBD); (2) HA inhibition depends on the number of galloyl substituents in a pGG analog; the best number is four; and when PGG binds with two HA trimers at their conserved receptor binding domains (loop 130, loop 220, and 190-α-helix), PGG acts as a molecular glue by aggregating viral particles so as to prevent viral entry into host cells (this was revealed via an *in silico* simulation on the binding of penta-galloyl-glucose (PGG) with HA). pGGs are also effective on a broad-spectrum influenza A subtypes (including H1, H3, H5, H7); this suggests that pGG analogs can be applied to most influenza A subtypes as a prophylactic against influenza viral infections.

## Introduction

The influenza A virus (IAV) is a single-stranded (−) RNA virus. It is the causative agent of annual seasonal influenza epidemics and occasional global pandemics; such epidemics are a leading cause of morbidity and mortality worldwide [1]. Global outbreaks of highly pathogenic avian influenza A H5N1 virus among poultry have caused sporadic human infections with a death rate of almost 60% (for all laboratory-confirmed cases) [2]. Recent studies have shown that laboratory-generated H5N1 viruses can be efficiently transmissible via air-borne droplets between ferrets (a model animal for human transmission); these findings have raised great concern regarding the emergence of H5N1 variants with high virulence (*i.e.*, of variants which have pandemic potentials for human-to-human transmission) [3]–[6]. On March 30, 2013, a novel avian influenza A H7N9 virus that caused human infections was identified in China [7]. The emergence of this new H7N9 bird flu virus caused severe illness and death in people (the mortality rate was more than 30%). Although there is no convincing evidence that the virus efficiently spreads among humans, there are still increasing infections reported continuously, which raised serious public health concerns worldwide [8].

Hemagglutinin (HA), a trimeric spike glycoprotein of the influenza virus, is responsible for binding to sialylated glycan receptors on the surface of host cells, thus inducing viral entry via endocytosis and followed by membrane fusion between the viral envelope and the endosome [9], [10]. There are at least 17 distinct HA antigenic subtypes (H1–H17) in influenza A viruses; 16 subtypes of HA have been identified from waterfowl reservoirs [11], and a new subtype, H17, has been identified from fruit bats in 2012 [12]. These subtypes are divided into two major phylogenetic groups [13]. Group 1 is composed of subtypes H1, H2, H5, H6, H8, H9, H11, H12, H13, H16, and H17. Group 2 includes subtypes H3, H4, H7, H10, H14 and H15 [14], [15]. HA is initially synthesized as a precursor, HA0. HA0 is proteolytically cleaved into HA1 and HA2 subunits. HA1 contains a receptor binding domain (RBD) in its globular head, which is located at the distal tip of each HA monomer. The RBD is formed with three conserved secondary structures: one helix (a 190-helix at the top of HA, consisting of residues 190–198) and two loops (a 130-loop, consisting of residues 135–138; and a 220-loop, consisting of residues 221–228) at the edges of the globular head of each HA monomer [16]–[18]. HA2 and both the N and C terminals of HA1 form a membrane-proximal stalk that mediates membrane fusion during viral entry [19]. Studies on HA suggest that avian and human influenza viruses appear to be distinct at the 220-loop (between residues 226 and 228 in the RBD) [20]. The HA proteins derived from avian influenza viruses bind specifically to the receptor α2,3-Gal-linked sialic acids (SA) (SAα2,3Gal), whereas human-adapted influenza viruses prefer to bind to the α2,6-Gal-linked SA (SAα2,6Gal). A shift from SAα2,3Gal-binding to SAα2,6Gal-binding specificity is a critical step in the adaptation of avian influenza viruses to human hosts, so as to cause pandemics. Indeed, four influenza pandemic strains, which occurred in 1918 (H1N1), 1957 (H2N2), 1968 (H3N2), and 2009 (H1N1), exhibited human-type receptor-binding specificity, although their HAs originated from non-human species. In humans, SAα2,6Gal is predominantly found in the upper respiratory tract, while SAα2,3Gal is also predominantly present in the lower respiratory tract [21]. Some mutant viruses displayed increased virulence in mice. This was likely the result of altered receptor specificities, which allowed the viruses to bind both SAα2,3Gal and SAα2,6Gal. These findings thus indicate that HA is an important determinant for pathogenicity [22].

Since HA is the major surface antigen of the influenza virus, anti-HA antibodies efficiently neutralize influenza viruses *in vitro* and *in vivo* by blocking HA-mediated virus attachment and cell entry. The most protective neutralizing antibodies are mainly directed against the globular head and interfere with HA-receptor docking [23]. Targeting HA at RBD may potentially combat a larger range of the strains and subtypes of influenza viruses. However, influenza viruses accumulate amino acid substitutions in the HA globular domain due to massive immune response selective pressure; this leads to RBD variants. The RBD of the HA protein is the major determinant of host switching, is responsible for receptor binding and viral entry, and mediates differences in virulence. The RBD therefore provides an attractive target for anti-IAV drugs and vaccines (that may potentially prevent divergent influenza viral strain infections). However, the development of HA inhibitors is hindered by their subtype dependency. Small molecular HA inhibitors have been reported [24]–[27]. But problematically, no licensed HA inhibitor drug is clinically available for influenza viruses yet. The FDA-approved anti-influenza drugs are M2 ion channel protein blockers (*e.g.*, amantadine and rimantadine) and neuraminidase inhibitors (*e.g.*, zanamivir and oseltamivir) [28]. The emergence of drug-resistant issues and new influenza virus strains urges us to develop new drugs with diverse mechanisms of action.

We previously reported that penta-galloyl-glucose (PGG), a poly-galloyl-glucose (pGG) analog, demonstrated anti-influenza virus activity by directly interacting with the HA protein. The cytotoxicity of PGG was evaluated on MDCK cells (EC50∶31.48±4.60 μM). No significant cytotoxicity was observed at the active concentration of PGG (EC50∶2.51±0.31 μM) [29], [30]. This study aims to elucidate, at the molecular level, the mechanism of action of pGG analogs (including PGG) as HA inhibitors through in vitro and in silico comparisons of quantitative structure-activity relationships (QSAR).

## Materials and Methods

### Compounds, Proteins, Cells, and Viruses

Gallic acid (GA) was purchased from Sigma-Aldrich. TGG and PGG were isolated from *Phyllanthus emblica Linn* with a purity >98% (32). EA, EGCG, and tannic acid were obtained from the Guangdong small molecule tangible library (GSMTL) [31] of Sun Yat-sen University. Recombinant influenza A proteins of four HA subtypes were purchased from Sino Biological Inc., including A/Puerto Rico/8/1934 (H1N1), A/California/04/2009 (H1N1), A/Netherlands/219/03 (H7N7), and A/Anhui/1/2005 (H5N1). MDCK cells were cultured in minimum essential medium (Invitrogen, USA) supplemented with 10% FBS (Cell culture bioscience, USA) and antibiotics (100 U/ml penicillin and 100 μg/ml streptomycin, Nacalai tesque, Japan). Influenza viruses A/WSN/33 (H1N1), A/PR8/34 (H1N1) and A/HK/8/68 (H3N2) were propagated in 10-day-old embryonated chicken eggs.

### Hemagglutination Inhibition Assay

The assay was performed by using chicken red blood cells (RBCs) with standard procedures. Each compound solution (25 μl), after a two-fold serial dilution in PBS, was mixed with an equal volume of influenza virus solution. The mixture was incubated in a 96-well plate for 1 hour at 4°C. Then, it was mixed with 50 μl of 1% chicken RBC suspension and incubated for 30 minutes at room temperature.

### Plaque Reduction Assay

The influenza viruses (A/WSN/33(H1N1), A/PR/8/34(H1N1), and A/HK/8/68(H3N2)) were pre-incubated with test samples (PGG, TGG, GA) diluted in MEM on ice for 1 hour. The mixture was then serially diluted and added to MDCK cells in 6-well plates and incubated for 1 hour at 37°C. After washing twice with PBS (−), cells were overlaid with MEM containing 0.8% (w/v) low melting agarose, 0.1% (w/v) BSA, 1% (v/v) vitamins, and 0.03% (w/v) glutamine. After 3 days of incubation, cells were fixed with ethanol: acetic acid (v/v = 1∶1) for 1 hour at room temperature. After removal of the overlaying agarose gel, the cells were stained with 2.5% (w/v) Amino Black 10B. The plaques were counted by visual examination. Means and standard deviations were calculated from three independent experiments.

### Immunofluorescence Microscopy

The influenza virus A/WSN/33(10^5^PFU) was incubated with either pGGs (10 μM) or DMSO (0.1%, v/v) on ice for one hour. The MDCK cells were then inoculated with the viral mixtures at 37°C for one hour. Then the cells were fixed and stained (for immunofluorescence) at two hours post-infection and analyzed via fluorescence microscopy. The cells were co-stained with anti-nucleoprotein antibody and Hoechst 33342.

### Surface Plasmon Resonance (SPR)

This experiment was performed with the ProteOn XPR36 Protein Interaction Array System (BIO-RAD) using a GLH sensor chip. The chip contains a 6×6 perpendicularly intersecting channel array. Four HA subtypes (*i.e.*, A/Puerto Rico/8/1934 (H1N1), A/California/04/2009 (H1N1), A/Netherlands/219/03 (H7N7), and A/Anhui/1/2005 (H5N1)) with the same electrophoresis mobility shift were immobilized via the amine-coupling method on the vertical channel, while poly-galloyl-glucose analogs were injected, as analytes, through the horizontal channel. According to the manufacturer’s instructions, a mixture of N-hydroxysulfosuccinimide (sulfo-NHS, 33 μM) and 1-ethyl-3- (3-dimethylamino-propyl) carbodiimide hydrochloride (EDAC, 133 μM) in water was injected (flow rate 25 μl/min) to activate the alginate polymer modified surface (GLH chip). After chip activation, four channels were coupled with the four HA subtypes (which were dissolved in 10 μM sodium acetate buffer pH 4.0), while one reference channel was capped with ethanolamine (1 M, pH 8.5). After the coupling step, the remaining surface of the coupled channel of the chip was deactivated with 1 M ethanolamine (pH 8.5). After extensive washing with running buffer, the chip was ready for binding studies. For analytes, the typical injection time was 300 s, at a rate of 25 μl/min. After association of analytes with ligands, running buffer was injected (for ≥300 s at 25 μl/min) to dissociate the bonded analyte. A regenerated chip surface is needed to get the surface plasmon resonance signal back to the baseline response level; this is accomplished via a buffer and sodium hydroxide solution. The RU was measured by subtracting the reference signal of the protein unlabeled channel from the double reference signal of the vehicle channel. The maximal RU is specified as R_max_; R_max_ indicates the maximal binding capacity of an immobilized protein. Then kinetic constants of each sensorgram (K_a_, K_d_) were calculated by fitting the binding model (Langmuir) to sensorgram curves using ProteOn Manager Software. Immobilization buffers were 10 mM sodium acetate buffer (pH 4.0). The running buffer was PBS buffer (136.75 mM NaCl, 2.69 mM KCl, 24.58 mM Na_2_HPO_4_, and 1.47 mM KH_2_PO_4_ pH 7.4) plus 0.1% DMSO.

### Transmission Electron Microscopy (TEM)

For the TEM study, purified influenza virus A/WSN/33 (10^6^ PFU) was mixed 1∶1 with either PGG, DMSO or TritonX-100 (Calbiochem, USA), diluted in PBS, and incubated on ice for 30 min. The final concentrations of PGG, DMSO or TritonX-100 were 25 μM, 0.1% (v/v), and 0.5% (v/v), respectively. After incubation, each mixture was added onto a 200-mesh and was removed 2 minutes later. Samples were then stained with 2% phosphotungstic acid for 20 seconds and subjected to detection using the JEM-1400 Transmission Electron Microscope (JEOL, Japan).

### Atomic Force Microscopy (AFM)

Purified A/WSN/33 (10^6^ PFU) was pre-incubated with either a tested compound or DMSO (Sigma-Aldrich, USA) diluted in PBS for 1 hour on ice, and allowed to deposit on a coverslip; the final concentrations of each compound or DMSO mixture were 50 μM and 0.1% (v/v), respectively. After washing, the deposited viruses were fixed with 2.5% glutaraldehyde for 15 minutes, and then dried with nitrogen gas and detected via the BioScope Catalyst Atomic Force Microscope (Bruker, USA).

### Molecular Modeling

Structure preparations were done with the molecular modeling package, MOE 2011.10 (Chemical Computing Group, Inc. Montreal, Canada). The initial structures of HAs were derived from known crystal structures, with some necessary modifications (in accordance with BLAST analyses) that accounted for the differences between the sequences of bioassay strains and crystal structures (see the Supplementary Table S1). The ligand structures were generated from MOE, and their geometry was optimized with the HF/6-31G(d) model in Gaussian 09 [32].The multiple binding structures were built in Discovery Studio v2.55 (Accelrys Inc.) using in-house Perl scripts. Docking programs were evaluated and selected by re-docking experiments as described in Supplementary Information. The results of re-docking are shown in Fig. S1.

All MD simulations were performed using AMBER 11 [33], [34]. The partial atomic charges of the ligands were calculated via the Gaussian 09 program by using the Hartree-Fock method with the 6-31G(d) basis set; the Antechamber program was then used to fit the restricted electrostatic potential (RESP) and assign the GAFF force field parameters [35]. For the protein receptors, the AMBER ff03 force field was used. The complexes were neutralized by adding sodium/chlorine counter ions, and solvated in an octahedral box of TIP3P [36] water molecules with solvent layers 10 Å between the box edges and solute surface. The SHAKE [37] algorithm was used to restrict all covalent bonds involving hydrogen atoms with a time step of 2 fs. The particle-mesh Ewald (PME) method [38] was performed to treat long-range electrostatic interactions. For each system, three steps of minimization were performed before the heating step. First, all atoms in the receptor-ligand complex were restrained with constraints of 50 kcal/(mol·Å^2^) whereas solvent molecules were not restrained. This step contained 2000 cycles of steepest descent minimization and 2000 cycles of conjugated gradient minimization. Then, all heavy atoms were restrained with constraints of 10 kcal/(mol·Å^2^) during the minimization steps, which consisted of 2500 cycles of steepest descent minimization and 2500 cycles of conjugated gradient minimization. For the final step, 5000 cycles of steepest descent minimization and 5000 cycles of conjugated gradient minimization were carried out without restraint. After minimizations, the whole system was first heated from 0 K to 300 K in 50 ps using Langevin dynamics at a constant volume and then equilibrated for 400 ps at a constant pressure of 1 atm. A weak constraint of 10 kcal/(mol·Å^2^) was used to restrain all the heavy atoms in the receptor-ligand complexes during the heating and steps. Finally, periodic boundary dynamics simulations were carried out for the whole system with NPT conditions (*i.e.*, at a constant pressure of 1 atm and constant temperature of 300 K in the production step). In the production phase, the complex of pGG and HA monomer was simulated for 10 ns; and the complex of pGG and HA dimer was simulated for 100 ns. The MM/PBSA method [39], [40] was used to calculate the binding energies (Supplementary Information). The root-mean-square deviation (RMSD) and B-factor that commonly used for assessing the MD quality were also calculated (Fig. S2).

### Electrophoretic Mobility Shift Assay

This assay was carried out to verify the agglutination effect of PGG on HA. Recombinant influenza A HA proteins were incubated on ice with excessive concentrations of either PGG or GA for 30 min. After incubation, the samples were mixed with loading buffer (100 mM Tris-Cl pH8.0, 40% Glycerol, 0.5% Coomassie Blue G-250(w/v)) and loaded into sample wells of nondenaturing 4%–15% Mini-PROTEAN® TGX™ Precast Gel (Bio-Rad). 0.002% Coomassie Blue G-250(w/v) was added to the Tris-Glycine cathode buffer. Electrophoresis was performed in the native PAGE gel at a constant current of 15 mA for 20 min for the stacking gel, and at 25 mA for 90 min for the separating gel (with ice-cooling running buffer). After electrophoresis on the native PAGE, the gels were then stained with the Silver Stain Kit (Thermo Scientific). HA (H7N7) and HA (H5N1), due to severe self-aggregation, can hardly migrate into the stacking gel even in the absence of PGG. Thus, 2% SDS was added in the sample loading buffer to improve the solubility of HA. As for HA (H1N1/PR8) and HA (H1N1/09), the self-aggregation issue is less severe and tolerable for electrophoresis.

## Results

### pGG Analogs Significantly Inhibit Virus-induced Hemagglutination

**Figure 1:**
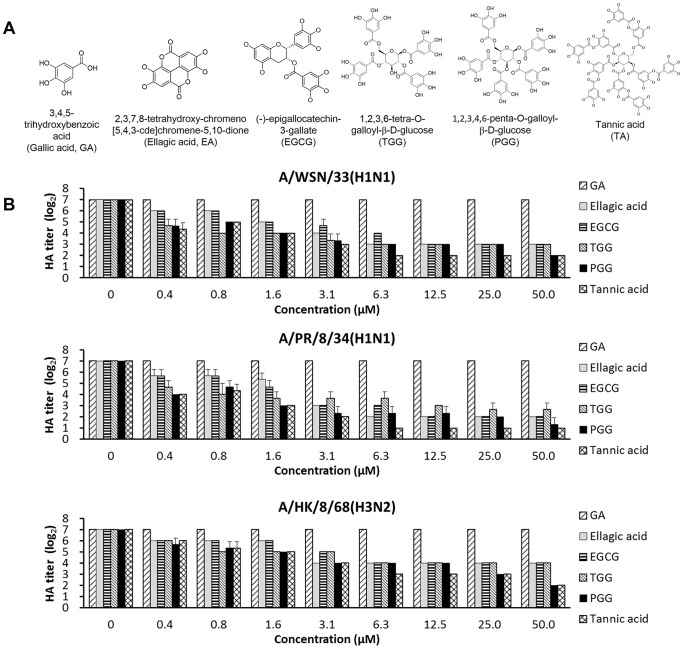
pGG analogs and their hemagglutination inhibition activities. (A) Chemical structures of pGG analogs. (B) pGG analogs inhibit the virus-induced hemagglutinations of virus subtypes A/WSN/33(H1N1), A/PR/8/34(H1N1), and A/HK/8/68(H3N2). Each compound solution (25 μl) with two-fold serial dilution in PBS was mixed with an equal volume of influenza virus solution. The mixture was incubated in a 96-well-plate for 1 hour at 4°C, and then was mixed with 50 μl of 1% chicken erythrocyte suspension and incubated for 30 minutes at room temperature. HA titers were determined. Three independent experiments were performed in triplicate.

PGG exhibits an anti-viral activity against influenza A viruses by inhibiting virus-induced hemagglutination and viral entry [29]. To elucidate the mechanism of action, we selected gallic acid (GA) and a number of pGG analogs containing different numbers of galloyl substituents (**Fig. 1A** **)** to study the relationship between HA inhibitory activities and pGG analog structures.

Hemagglutination inhibition assays reveal that GA cannot inhibit virus-induced hemagglutination; other pGG analogs, however, can significantly inhibit virus-induced hemagglutination in a dose-dependent manner (**Fig. 1B**). The inhibitory effects of pGG analogs on the virus-induced hemagglutination were measured in EC_50_ (effective concentration, 50%) and shown in **Table 1**. 1,2,3,6-Tetra-O-galloyl-beta-D-glucose (TGG), PGG, and tannic acid (TA), which possesses four or more galloyl substituents, demonstrate higher activities than that of Ellagic acid (EA) and (−)-epigallocatechin-3-gallate (EGCG). These is a clear qualitative relationship between HA inhibition and the number of galloyl substituents in pGG analogs. In addition, these pGG analogs show higher inhibitions of influenza A/H1N1, compared to inhibitions of influenza A/H3N2.

**Table 1:** Inhibitory effects of poly-galloyl-glucose series on virus-induced hemagglutination.

	EC_50_ (μM)					
	GA	EA	EGCG	TGG	PGG	TA
**A/WSN/33 (H1N1)**	>50	0.446±0.000	0.430±0.001	0.001±0.001	0.005±0.002	0.006±0.003
**A/PR/8/34 (H1N1)**	>50	0.418±0.370	0.307±0.260	0.001±0.002	0.004±0.004	0.002±0.002
**A/HK/8/68 (H3N2)**	>50	0.781±0.000	0.731±0.000	0.195±0.000	0.202±0.206	0.272±0.139

The data shown are the means ± standard deviations (SD) of two to three independent tests. The activity is expressed as the EC_50_ defined as the compound concentration producing 50% inhibition of hemagglutination induced by influenza virus, as estimated by hemagglutination test.

### pGG Analogs Inhibit Viral Infectivity and Virus Entry

**Figure 2:**
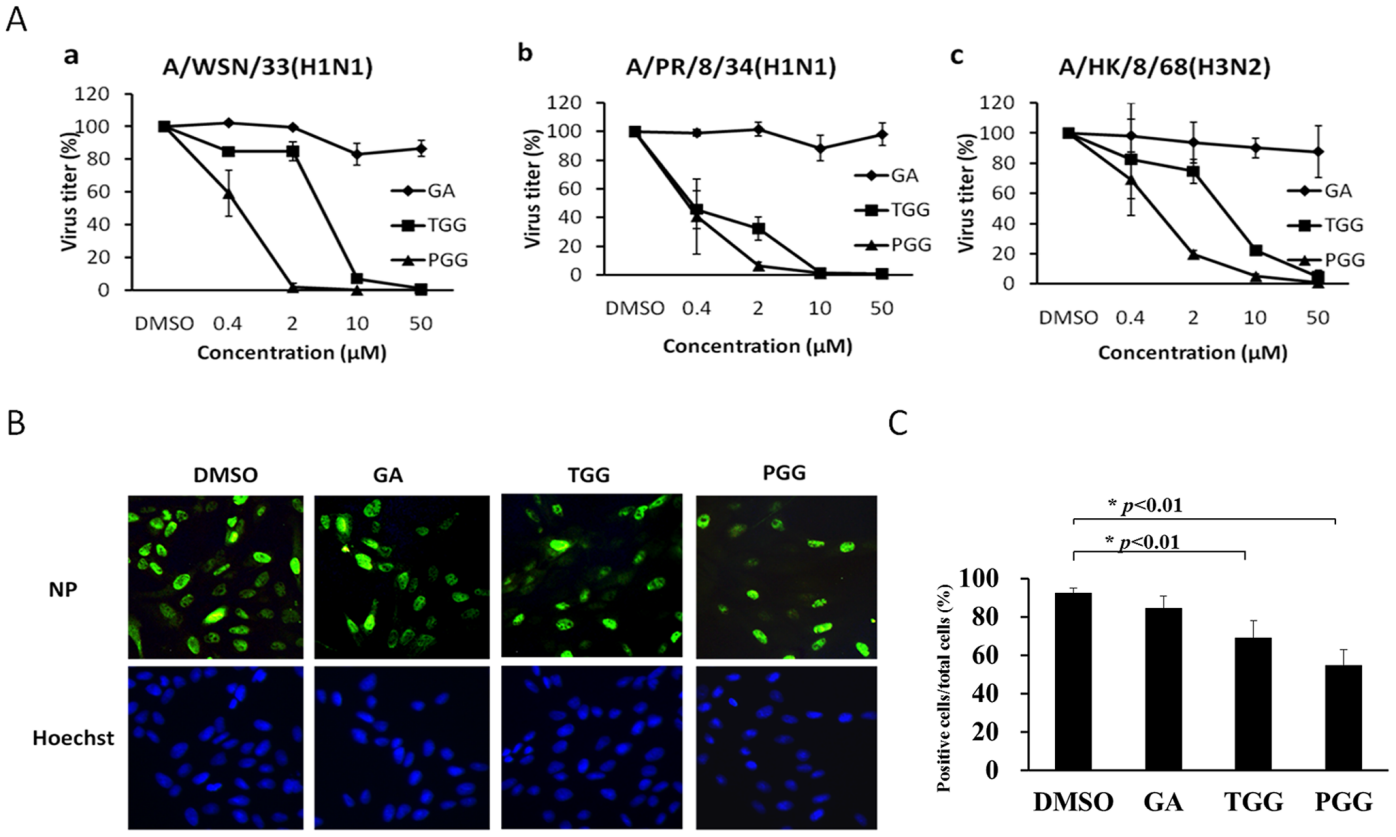
pGG analogs inhibit viral infectivity and block viral entry. (A) The viral infectivity was titrated directly via a plaque assay on MDCK cells. Values represent the mean of three independent experiments, and error bars show the standard deviation of the mean. (B) Influenza virus A/WSN/33(10^5^PFU) was incubated with the compounds (10 μM) or DMSO (0.1%, v/v) on ice for one hour and then inoculated to MDCK cells at 37°C for one hour. Cells were fixed and immunofluorescence stained at two hours post-infection and analyzed by fluorescence microscopy. Cells were co-stained with anti-nucleoprotein antibody and Hoechst 33342. (C) The number of positive cells (green) and the total number of cells were counted, and the ratio of positive cells to total cells was calculated. 500 cells were included for each group. The data was statistically analyzed using the Student’s t test. *p<0.01.

A plaque reduction assay was performed to determine the effects of GA, TGG and PGG on viral infectivity. As shown in **Fig. 2A**, GA does not show inhibitory effect on virus titers of three distinct strains, but TGG and PGG significantly reduced the virus titers of the strains in dose-dependent manner. The effects of pGG analogs on virus entry were also confirmed by detecting intra-nuclear accumulation of virus nucleoprotein (NP). The accumulation is an early event during IAV entry. As shown in **Fig. 2B**, viral NP was detected in the nucleus of GA-treated cells as well as the DMSO-treated cells (vehicle control) at two hours post-infection, whereas, significantly decreased in the number of nucleoprotein-positive nucleus in TGG-treated and PGG-treated cells (**Fig. 2C**). These results indicate that viral infectivity and viral entry are significantly inhibited by TGG and PGG, but not GA.

### pGG Analogs Cross-linking Hemagglutinin Molecules

**Figure 3:**
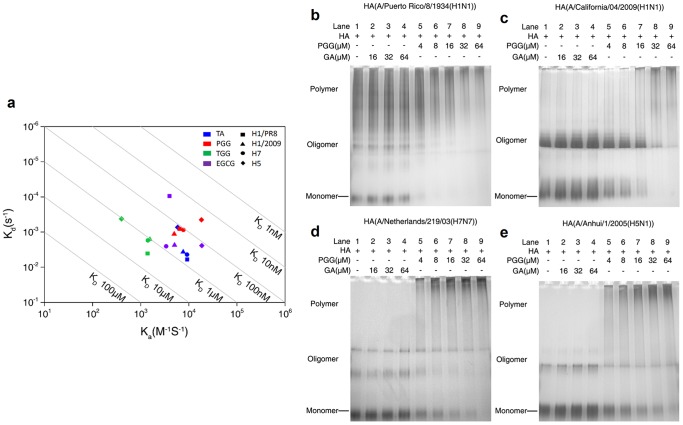
pGG analogs directly bind and aggregate HA molecules. (a) SPR results of the associations and dissociations of pGG analogs and HA molecules. K_a_ and K_d_ were calculated by fitting the binding model (Langmuir) to sensorgram curves using ProteOn Manager Software. (c-f) Native PAGE and silver stains reveal the effects of binding of PGG on the mobility of HA after pre-incubation with or without PGG. Four subtypes of purified proteins of HA (A/Puerto Rico/8/1934 (H1N1), A/California/04/2009 (H1N1), A/Netherlands/219/03 (H7N7), and A/Anhui/1/2005 (H5N1)) were tested and shown as panels (b), (c), (d), and (e), respectively.

Surface plasmon resonance (SPR) experiments were conducted to inspect the association or dissociation of pGG analogs and hemagglutinin molecules. After immobilizing purified recombinant monomeric hemagglutinin derived from IAV subtypes (A/Puerto Rico/8/1934 (H1N1), A/California/04/2009 (H1N1), A/Netherlands/219/03 (H7N7), and A/Anhui/1/2005 (H5N1)) on the chip, we analyzed the interactions of pGG analogs and hemagglutinin molecules by flowing pGG analogs through biosensor channels. The ligand-receptor interactions were measured by the sensorgram, which was plotted as response units (RUs) versus time (Supplementary Information). The K_a_ (association constant) and K_d_ (dissociation constant) were derived from the sensorgram. The K_a_ and K_d_ are depicted in **Fig. 3a**, which indicates that the affinity constants (K_D_) of pGG analogs (TA, PGG, TGG, and EGCG) for hemagglutinin range from 10 nM to 1 μM. This proves that pGG analogs directly interact with hemagglutinin molecules. No significant interactions with HAs were detected for GA.

Native polyacrylamide gel electrophoreses (PAGE) experiments demonstrate that the concentration of HA monomer or oligomer decreases while the concentration of the incubated PGG increases. Moreover, the concentration of HA polymer increases while the concentration of the incubated PGG increases. The phenomena were also observed in the PGG and other HA subtypes (A/Puerto Rico/8/1934 (H1N1), A/California/04/2009 (H1N1), A/Netherlands/219/03 (H7N7), and A/Anhui/1/2005 (H5N1) PAGE experiments (**Figs. 3b–3e**). On the other hand, the phenomena were not observed in GA and HA mixtures.

The above experimental results confirm that pGG analogs directly interact with HA molecules, and cause aggregation. It is worth noting that the H7N7 used in our experiments is a highly similar homolog of H7N9, which accounts for a new outbreak in China.

Three *in silico* experiments were conducted to model PGG aggregating hemagglutinin oligomers. Three models were built based upon hemagglutinin crystal structures (PDB codes: 1RVZ) with additional 50 ns MD simulations. As shown in **Fig. S5**, model A, in which a PGG molecule aggregates two trans-oriented hemagglutinin trimers, has a significantly lower binding energy than the ones of other models (**Table S2**). Actually, PGG can aggregate two, three, and more hemagglutinin trimers, which further form hemagglutinin oligomers, and eventually hemagglutinin polymers (**Figs. 3b–3e**).

**Figure 4:**
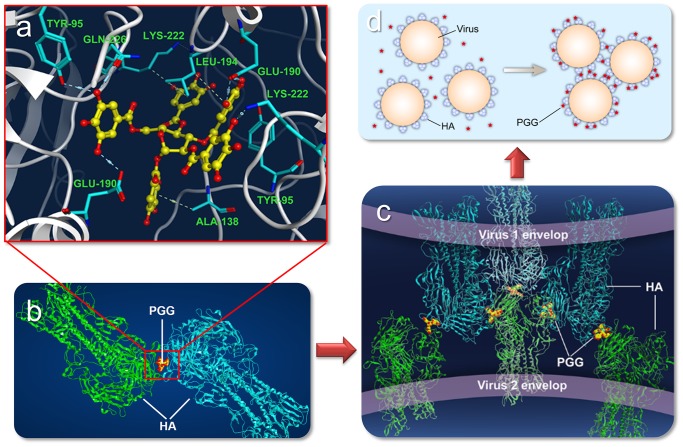
Proposed process of PGG aggregating the flu-virus particles. (a) PGG binds with two different HA trimers through H-bonds and π stacking interactions at the RBDs. (b) A pair of HA trimers are aggregated by a PGG molecule. The paired HA trimers are further aggregated to form HA oligomers. (c) The HA polymers are formed from the PGG-induced HA oligomers. (d) The flu-virus particles are aggregated by PGG.

The process of PGG aggregating the flu-virus particles is proposed in **Fig. 4**: PGG binds with two hemagglutinin trimers at their receptor binding domains (loop 130, loop 220, and 190-α-helix) (**Figs. 4a and 4b**); consequently, PGG-induced hemagglutinin oligomers are further aggregated to form PGG-induced hemagglutinin polymers (**Fig. 4c**). Eventually, the flu-virus particles are aggregated by PGG molecules.

### pGG Analogs Agglutinate Virus Particles

**Figure 5:**
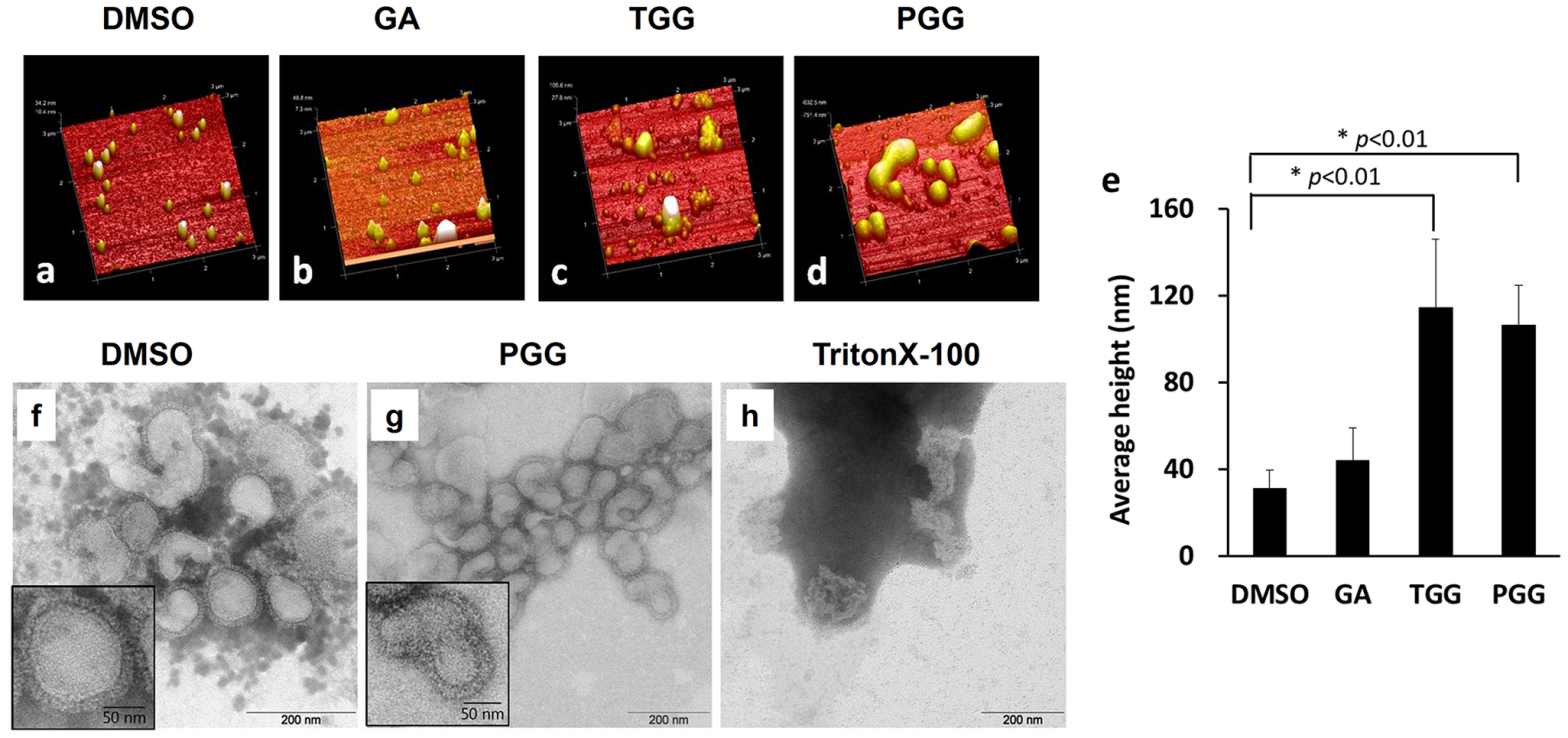
AFM and TEM experimental results confirm that pGG analogs agglutinate influenza virus particles without significantly disrupting the flu viral membrane structures. (a−e): AFM experimental results. Purified influenza virus A/WSN/33was pre-incubated with either DMSO, PGG, or TritonX-100 for 60 minutes on ice. The AFM image size is 3 μm from 80 scans. The data was statistically analyzed using the Student’s t test. *p<0.01. (f-h): Influenza virus A/WSN/33 (10^6^ PFU) was incubated with either PGG, TritonX-100, or DMSO for 60 minutes on ice. Boxed areas with the full line are shown at a higher magnification.

Atomic force microscopy (AFM) experiments were performed to confirm that pGG analogs agglutinate viral particles through binding hemagglutinin. The AFM images of virus, as shown in **Figs. 5a∼5d**, were taken after the viruses were treated with DMSO (the vehicle control), GA, TGG, and PGG. The flu virus particles without pGG analog treatments (**Fig. 5a**), as well as the ones treated with GA (**Fig. 5b**), are dispersive; the borders of virions are clear. However, the viral particles treated with TGG (**Fig. 5c**) and PGG (**Fig. 5d**) are significantly aggregated; the sizes of the particles are significantly larger than the ones in **Figs. 5a and 5b**. The heights of TGG-treated and PGG-treated virions were significantly greater than that of DMSO-treated virions (**Fig. 5e**).

Transmission electron microscopy (TEM) experiments (**Figs. 5f∼5h**) indicate that the membrane structures of the virus particles treated with PGG remain undisrupted (**Fig. 5g**), and are similar to the membrane structures without pGG analog treatment (**Fig. 5f**). For comparison purposes, the destroyed membrane structures of the virus particles are shown in **Fig. 5h** (TritonX-100 was used as virion-disrupted control). The surface glycoprotein spikes can be found in both the DMSO-treated virus and in the PGG-treated virus (**Figs. 5f and 5g**). The PGG-treated virions are clumped, but the viral envelope membrane is not disrupted (**Fig. 5g**).

The results of both AFM and TEM experiments suggest that pGG analogs agglutinate influenza virus particles without significantly disrupting the viral membrane structures.

### pGG Analogs Bind to the Receptor-binding Domain of Hemagglutinin

**Figure 6:**
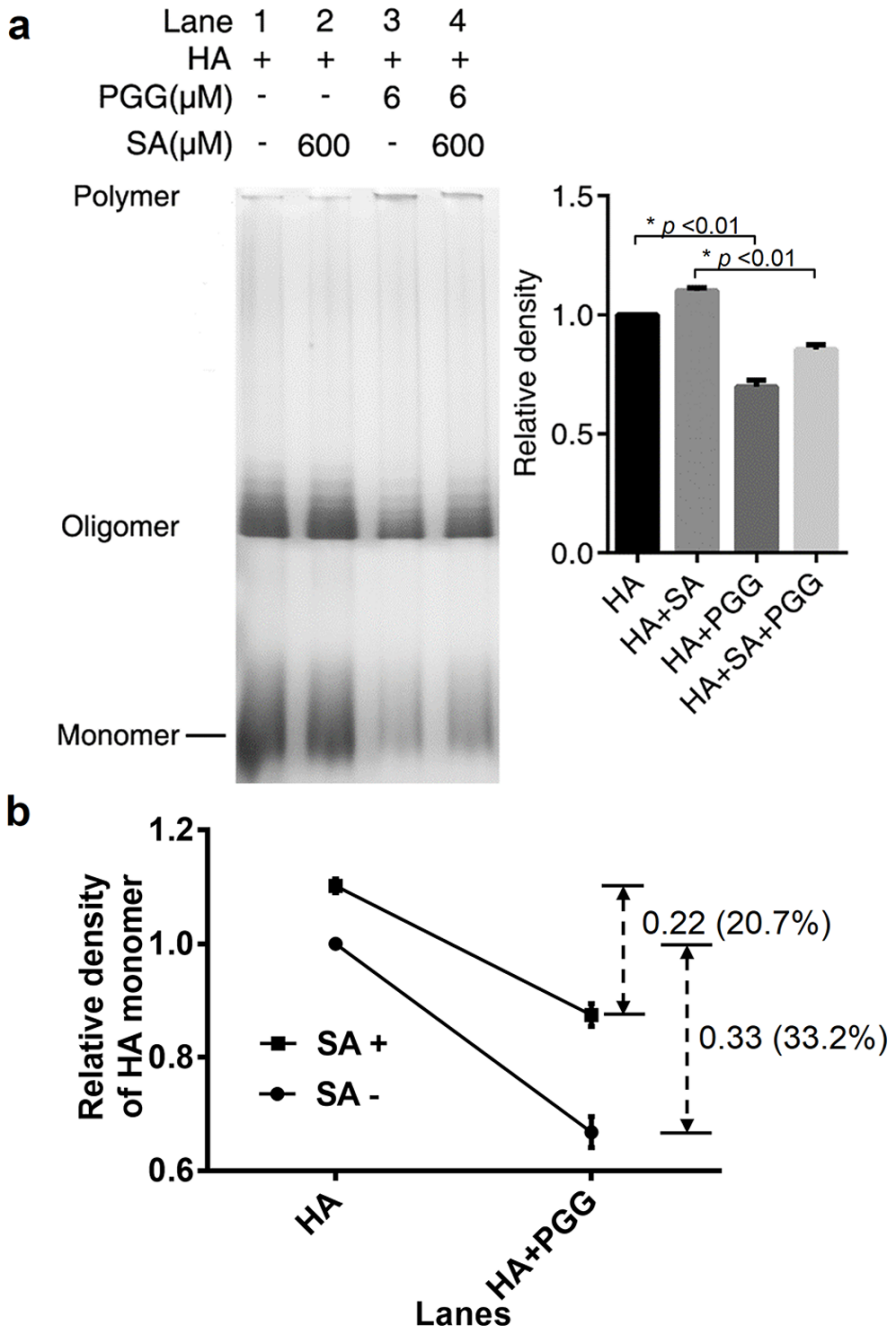
SA competitively inhibits PGG-induced HA aggregation. (a) The effects of SA on the electrophoretic mobility shift of HA and HA incubated with PGG. The relative density of HA monomer is statistically analyzed using the Student’s t test. *p<0.01 (b) The quantitative calculation of the decrease of HA monomers and oligomers with or without SA.

A competition binding assay was performed to determine whether PGG directly binds the hemagglutinin receptor binding domain (RBD). Sialic acid (SA), the substrate of hemagglutinin, was used as a competitive agent with PGG in the assay. SA was incubated with the hemagglutinin derived from influenza A/California/04/2009(H1N1) prior to PGG addition. The grayscale in a lane of **Fig. 6a** represents the concentration of HA monomer or oligomer. As shown in **Fig. 6a**, the assays results suggest that 600 μM SA reduces hemagglutinin aggregation (Lane 2); 6 μM PGG increases hemagglutinin aggregation (Lane 3); Simultaneous incubation of hemagglutinin with both 600 μM SA and 6 μM PGG still increases hemagglutinin aggregation (Lane 4), but the increases are much less comparing with Lane 3. To quantitatively estimate the SA effect on PGG binding activity, we have measured HA monomer concentration decrease by detecting the native page band gray scale (Fig. 6). With the presence of SA, the HA aggregation decreases from 0.33(33.2%) to 0.22 (20.7%). This is probably due to SA has competitive binding with HA. Thus, we conclude that PGG analogs bind to the hemagglutinin receptor-binding site, which is usually bound with SA.

### pGG Analogs Bind to Hemagglutinin through Hydrogen-bonds and π-σ Stacking

To study the binding modes of pGG analogs on hemagglutinin, molecular docking, molecular dynamics (MD) simulations, and binding affinity calculations were performed on six receptor-ligand complexes: H1N1/PR8 and PGG, H1N1/PR8 and TGG, H1N1/PR8 and EGCG, H1N1/PR8 and EA, H1N1/WSN and PGG, and H3N2/HK and PGG. The calculated binding energy results are listed in Table S3.

Energy favorable binding modes of the pGG analogs and H1N1/PR8 hemagglutinin complexes are presented in **Figs. S3a∼S3d** in the supplementary material. The receptor binding domain (RBD) has three conserved regions (loop 130, loop 220, and 190-α-helix). PGG, TGG, and EGCG interact with all three conserved regions through hydrogen bonds (H-bonds) and π-stacking. However, EA only interacts with the two conserved regions (loop 220 and 190-α-helix) through hydrogen binding. These binding modes are consistent with the experimental binding affinities (**Fig. S6**).

The MD simulation results suggest that the activities of pGG analogs depend on the number of galloyl substituents. Increasing the number of galloyl substituents will increase the inhibition. When the number of galloyl substituents reaches four, the inhibitory activity is close to the maximum. PGG has five galloyl substituents (**Fig. S3a**), and interacts with loop 130 through a σ-π stacking at ALAs 137, 138; H-bonds at the backbone atoms interact with loop 220 at GLN 226 and ARG 224; PGG also interacts with the 190-α-helix through H-bonds with GLU 190. Similarly, TGG interacts with loop 130 through H-bonds at backbone atoms and σ-π stacking at ALA 137 and VAL 134; TGG also interacts with loop 190 with H-bonds at GLU 190, and interacts with loop 220 through GLN 226 and ALA 227 (**Fig. S3b**). EGCG also interacts with the three conserved regions through H-bonds with GLU 190 and via σ-π stacking with LEU 194 (190-α-helix); EGCG also interacts via a hydrogen bond at GLN 226 (loop 220), and via backbone atoms forming H-bonds with VAL 135 (loop 130) (**Fig. S3c**). EA is small and ridged, and has only two galloyl groups. It cannot interact with the three binding areas of HA at the same time. Therefore, EA has a weaker binding affinity and hemagglutination inhibition (**Fig. S3d**). These *in silico* results are consistent with the *in vitro* hemagglutination inhibition assay data.

Comparing the binding modes of three ligand-receptor complexes (PGG-H1/PR8, PGG-H1/WSN, and PGG-H3/HK), we observed that PGG has a stronger binding affinity with H1, and a weaker binding affinity with H3 (**Figs. S4a** and **S4b**), because PGG has a lower electrostatic binding energy (ΔE_ele_) with H3. The solvent-accessible surface area [41] of the three hemagglutinin binding sites plots indicate that the solvent-accessible surface of the hemagglutinin for H1 homologs are different from those for H3 homologs (**Fig. S4c)**. HA has two subtype groups, H1 belongs to group 1, and H3 belongs to group 2. Compared with H1, H3’s binding pocket is deeper, and has greater solvent accessible surface area (SASA), which makes it more difficult for PGG to bind. H3 binding pocket is also bumpier (at loop 130 and loop 220) than the ones of H1, therefore, PGG binds to H3 in different way (**Figs. S4b and S4d)**.

**Figure 7:**
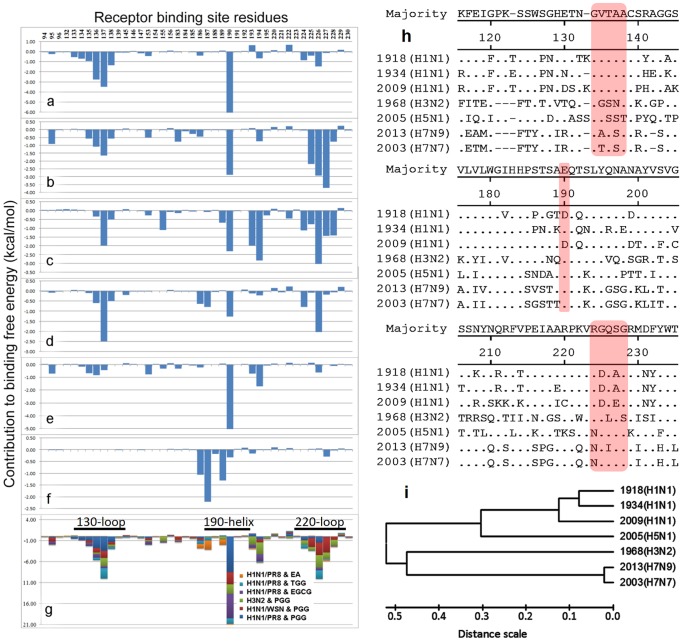
Energy decomposition analyses and sequence alignment analyses. (a−g) binding energy decomposition results for each modeled system. (a) PGG & H1N1/PR8, (b) PGG & H1N1/WSN, (c) PGG & H3N2/HK, (d) TGG & H1N1/PR8, (e) EGCG & H1N1/PR8, (f) EA & H1N1/PR8, (g) Bar chart combining the results from (a) ∼ (f). The residues included are those within 8 Å distance from the ligand binding site. The unit of the each residue’s contribution to total binding energy is kcal/mol. (h) Multiple sequence alignment of HA from A/Brevig Mission/1/1918(H1N1), A/Puerto Rico/8/1934(H1N1), A/California/07/2009(H1N1), A/HK/8/1968(H3N2), A/Vietnam/1203/2004(H5N1), A/HZ/1/2013(H7N9), and A/Netherlands/219/03(H7N7). The sequences were aligned using ClustalW algorithm by MegAlign (LaserGene v7.1, DNASTAR Inc.) (i) Phylogenetic tree of hemagglutinin sequences used for sequence alignment.

Energy decomposition analyses were conducted on the six ligand-receptor complexes (PGG-H3N2/HK, PGG-H1N1/WSN, PGG-H1N1/PR8, TGG-H1N1/PR8, EGCG-H1N1/PR8, and EA-H1N1/PR8) to identify the residues that make major contributions to binding affinities (**Figs. 7a–7f**). Extended analyses of another three systems (PGG-H1N1/2009, PGG-H5N1, PGG-H7N9) are shown in **Fig. S7**. These systems show similar distribution patterns. The key binding residues are summarized in **Fig. 7g**. These residues can be divided into three groups located at the three conserved regions: group I (residue 134–138) for loop 130, group II (residue 224–228) for loop 220, and group III (residue 190) for the 190-α-helix. pGG analogs have interactions with most of these key residues. This phenomenon leads pGG analogs to significantly inhibit hemagglutinin.

### Sequence Alignments show pGG Analogs Interact with Hemagglutinin Homologs at Conserved Regions

Seven representative influenza hemagglutinin sequences derived from H1, H3, H5, and H7 subtypes (A/Brevig Mission/1/1918 (H1N1), A/Puerto Rico/8/1934(H1N1), A/California/07/2009(H1N1), A/HK/8/1968(H3N2), A/Vietnam/1203/2004(H5N1), A/HZ/1/2013(H7N9), and A/Netherlands/219/03(H7N7)) were aligned at the RBD region using the ClustalW algorithm [42], and three conserved regions were highlighted (**Fig. 7h** **)**. Based upon the alignment result, a phylogenetic tree was generated (**Fig. 7i**). The phylogenetic tree indicates that the influenza hemagglutinin homologs can be divided into group I (H1 and H5) and group II (H3 and H7). The sequences from both groups are highly conserved at the RBD region (highlighted in **Fig. 7h** **)**, which can be selectively bound with pGG analogs (it was reported that residues 190 and 225 are critical determinants for the receptor-binding specificity of H1N1 HA [43]–[47]).

Sequence alignment analyses also demonstrate that the RBDs of 1934(H1N1/PR8), 1933(H1N1/WSN), H3N2, H5N1, H7N7, and H7N9 are highly conserved at residue 190 with glutamic acid (only two cases involve aspartic acid, which belongs to the same amino acid group). And pGG analogs have strong binding at this site through the carboxyl group of residue 190. On the other hand, pGG analogs are flexible enough to adjust themselves for possible minor mutations at residue 190. This is also supported by our SPR experiments, as shown in **Fig. 3a**.

The key residues at the 130-loop of RBD are basically conserved. There are a few mutations, and these involve mainly smaller sized residues (alanine, glycine, serine, and threonine). Again, pGG analogs can easily adjust themselves for binding at this domain with minor receptor changes. As shown in **Figs. S3 and S4**, pGG analogs can interact with the 130-loop mainly through backbone H-bonds and σ-π stacking without being disrupted by side chain changes.

The key residues at the 220-loop of RBD are well conserved with minor mutations. Residue 226 is glutamine for most of the subtypes (H1 and H5). pGG analogs bind with residue 226 via H-bonds. In case of H3N2, pGG analogs bind with residues 225 and 227 via Q226L.

Therefore, we propose that pGG analogs inhibit a number of hemagglutinin homologs due to they can bind at the RBDs that are conserved, and the RBDs are selective to the pGG scaffold.

## Discussion

The recognition of HA for its receptor is highly dependent on the structural features of HA conserved domains (despite the hyper-variability of the residues surrounding the HA receptor binding site). For HAs from the H1 virus, residues 190 and 225 at the RBD are the determinants of receptor binding specificity. The H1 HAs possessing E190 and G225 preferentially bind to α2–3 receptors in avian species; D190 and G225 bind to both α2–3 and α2–6 receptors in swine species, and D190 and D225 effectively bind to α2–6 receptors in *homo sapiens* [48]. Therefore, E190D and D225G mutations are critical for shifting from α2,3-glycan to α2,6-glycan recognition. For H2 and H3 HAs, the substitutions of Q226L and G228S (amino acid positions listed herein refer to H3 numbering) confer a complete switch from α2,3-glycan to α2,6-glycan binding. The Q226L mutation changes the binding preference from avian- to human-type in an avian H3 HA; the mutation is important for human receptor binding [4]. For H5N1, H7N2, and H9N2 viruses, the amino acid substitutions in the 220-loop may be critical for avian HAs to acquire human-type receptor specificity [5]. These amino acid changes in HA will confer the binding specificity for human-type receptors, and are required for cross-species transmission and human adaptation of avian influenza viruses (which lead to new pandemics). The subtype diversity and frequent changes in HA structure of influenza A strains enable the flu virus to cause large regional or global pandemic outbreaks. This makes it difficult to develop HA-specific anti-flu drugs.

Polyphenols can inhibit influenza viruses by interacting with the viral HA protein. The inhibition leads to virucidal activity by damaging the virions’ structural integrity; polyphenols aggregate virions to prevent the viruses from adsorbing to cells [49], [50].

In this study, our results for the first time demonstrate that pGG analogs bind with HA homologs (H1, H3, H5, and H7) at the highly conserved structural elements of the RBD of HA virus, through H-bond and σ-π stacking interactions. The RBD is responsible for the virus to recognize host cells. We have revealed that pGG analogs bind to HA resulting aggregating the flu viral particles without disrupting the viral envelope, and stop viral infectivity and entry.

Our AFM experiments proved that pGG analogs cause virion aggregations by clumping influenza viral particles. Consequently, influenza viral entry is blocked. The mechanism of action is different from the many sialic acid-containing agents that have been developed as HA inhibitors [25]. Besides effectively preventing HA from binding to receptors, pGG analogs also induce the aggregation of viral particles [25]. MD simulations also suggest that the molecules with multiple galloyl substituents tend to exhibit stable binding interactions with the three conserved binding sites (loop-130, helix-190, and loop-220) at the RBD of HA. The binding forms a triangular region that stabilizes the ligand-receptor complexes; such stabilization explains the strong ligand-receptor binding affinity. Each galloyl substituent in a pGG analog contributes to the binding affinity of PGG-HA complexes. Increasing the number of galloyl substituents in a pGG analog will increase the pGG analog’s inhibition of the flu virus. The pGG inhibition to HA is attributed to the star-shaped structural feature (a glycosyl core with a number of galloyl “fingers”). The “fingers” are capable of forming an antibody-like tertiary structure, which can simultaneously grip two separated HA trimers through their RBDs, neutralize divergent influenza HAs, and form stable pGG-HAs complexes. However, without the tertiary structure, gallic acid (*i.e.*, GA, a single galloyl “finger”), can only be self-docked into a HA binding pocket, but cannot grip two HA trimers. Therefore, the star-shaped tertiary structural feature is the key to the aggregating mechanism of pGG’s HA inhibition. Recent NMR studies on protein-PGG interaction also demonstrated that PGG binding involves both hydrophobic interactions and H-bonds [51]–[53]. Since pGG analogs consist of two simple chemoyls (glycosyl and galloyl), it is expected that new HA inhibitors can be identified from a star-shaped compound library assembled from glycosyl-like and galloyl-like chemoyls [54].

It has been reported that PGG can also enhance phagocytosis on dendritic cells; phagocytosis can play a pivotal role in initiating and controlling the T cell-dependent immune response [55]. Hence, it is expected that PGG binding virions can promote influenza virus clearance *in vivo*. Meanwhile, PGG exhibits anti-allergy activity by down-regulating mast cell surface FcεRI expression both *in vitro* and *in viv*o [56]. Since pGG analogs’ interaction with HA can be applied to most influenza subtypes, it is possible to use this strategy for prophylaxis against influenza viral infections.

## Supporting Information

Figure S1 **RMSD values of the top ten scoring poses obtained from docking results of MOE, CDOCKER, Ligandfit, Surflex and FlexX.**
(TIF)

Figure S2 **MD quality analyses by (a) RMSD values of receptor backbone, ligand and pocket residues are in black, red and green lines.** (b) B-factor values of HA before/after MD are in black/red lines. Due to space limitation, only one system’s result is shown to be representative.
(TIF)

Figure S3 **Representative binding modes of the most popular MD conformations from the trajectories of poly-galloyl-glucose analogs docked with HA/PR8.** The secondary structure of the proteins is in grey, PGG and its analogues are colored in yellow: (a) PGG (b) TGG (c) EGCG (d) EA, pocket residues within 5 Å distance from the ligand are colored in cyan. Hydrogen bonds are depicted in green dash lines.
(TIF)

Figure S4 **The most popular binding modes derived from MD simulations for PGG docked with (a) H1/WSN, and (b) H3/HK.** The secondary structure of the proteins is in grey, PGG is in yellow sticks, receptor residues within 5 Å distance from the ligand are represented in cyan sticks. Hydrogen bonds are depicted in green dash lines. The three HA proteins, H1/PR8(cyan), H1/WSN(magenta), H3/HK(green) were superimposed to compare the secondary structure variations upon PGG binding. (c) Time series of solvent-accessible surface area (SASA) of H1/PR8, H1/WSN and H3N2 pocket residues upon PGG binding, (d) side view of the HA pocket after superimposing the three HA proteins.
(TIF)

Figure S5 **Three proposed models of PGG gluing hemagglutinin oligomers.** HAs were represented by magenta and cyan ribbons. The red sphere marked the PGG binding site. The three models were built by creating the symmetric projection of the original HA and adding some rotation. (a) Trans-oriented model, created by centro symmetric projection (b) Cis-oriented model, created by mirror symmetric projection (c) Orthogonal-oriented model, created by 90° rotation of mirror symmetric projection model.
(TIF)

Figure S6 **Correlation plot of predicted and experimental binding energy.** The experimental binding energy is obtained by converting experimental K_D_ values using formula ΔG_binding_ = RTlnK.
(TIF)

Figure S7 **Binding energy decomposition results for another three systems.** (a) PGG & H1N1/2009, (b) PGG & H5N1, (c) PGG & H7N9.
(TIF)

Table S1 **Sequence identity between the HA strains for bioassay and the available HA 3D structures.**
(DOC)

Table S2 **Binding affinity calculation results for each conformation and each model.**
(DOC)

Table S3 **Various energy contributions to the binding energy for each complex system of pGG analogs vs. mono HA.**
(DOC)

Text S1 **Detailed methods for virus purification, redocking evaluation, BLAST, and binding energy calculations.**
(DOC)
