# Supplementary figures and images for: The Mechanism of Poly-Galloyl-Glucoses Preventing Influenza A Virus Entry into Host Cells
Source: PLoS One. 2014 Apr 9;9(4):e94392. doi: 10.1371/journal.pone.0094392 (PMC3981784; doi:10.1371/journal.pone.0094392)

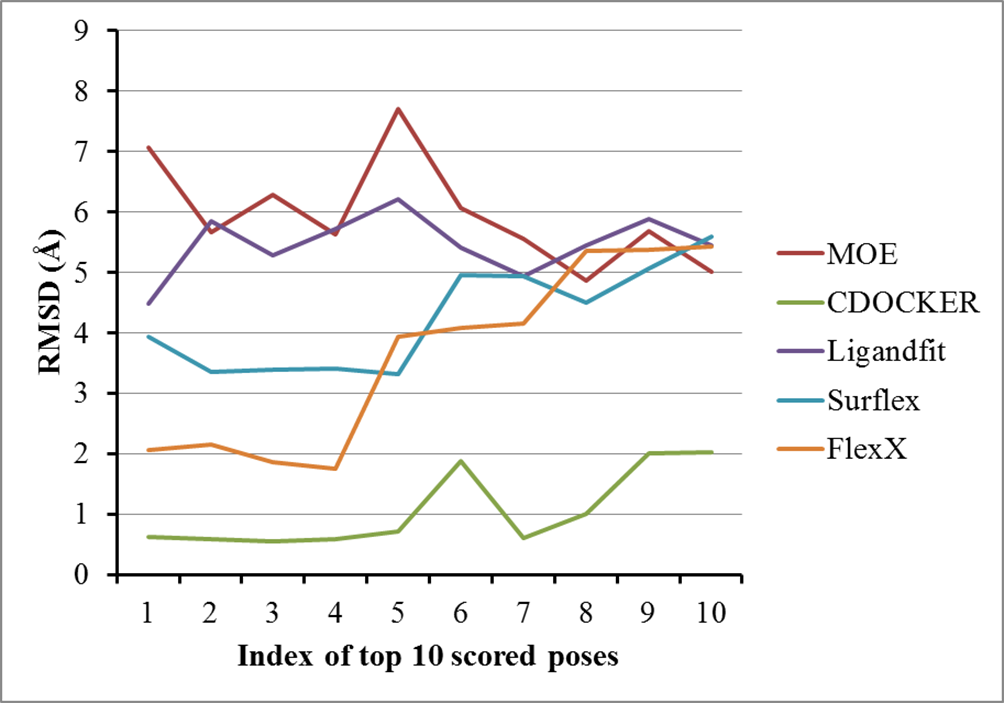

Supplement: Figure S1 — RMSD values of the top ten scoring poses obtained from docking results of MOE, CDOCKER, Ligandfit, Surflex and FlexX. (TIF) [file pone.0094392.s001.tif]

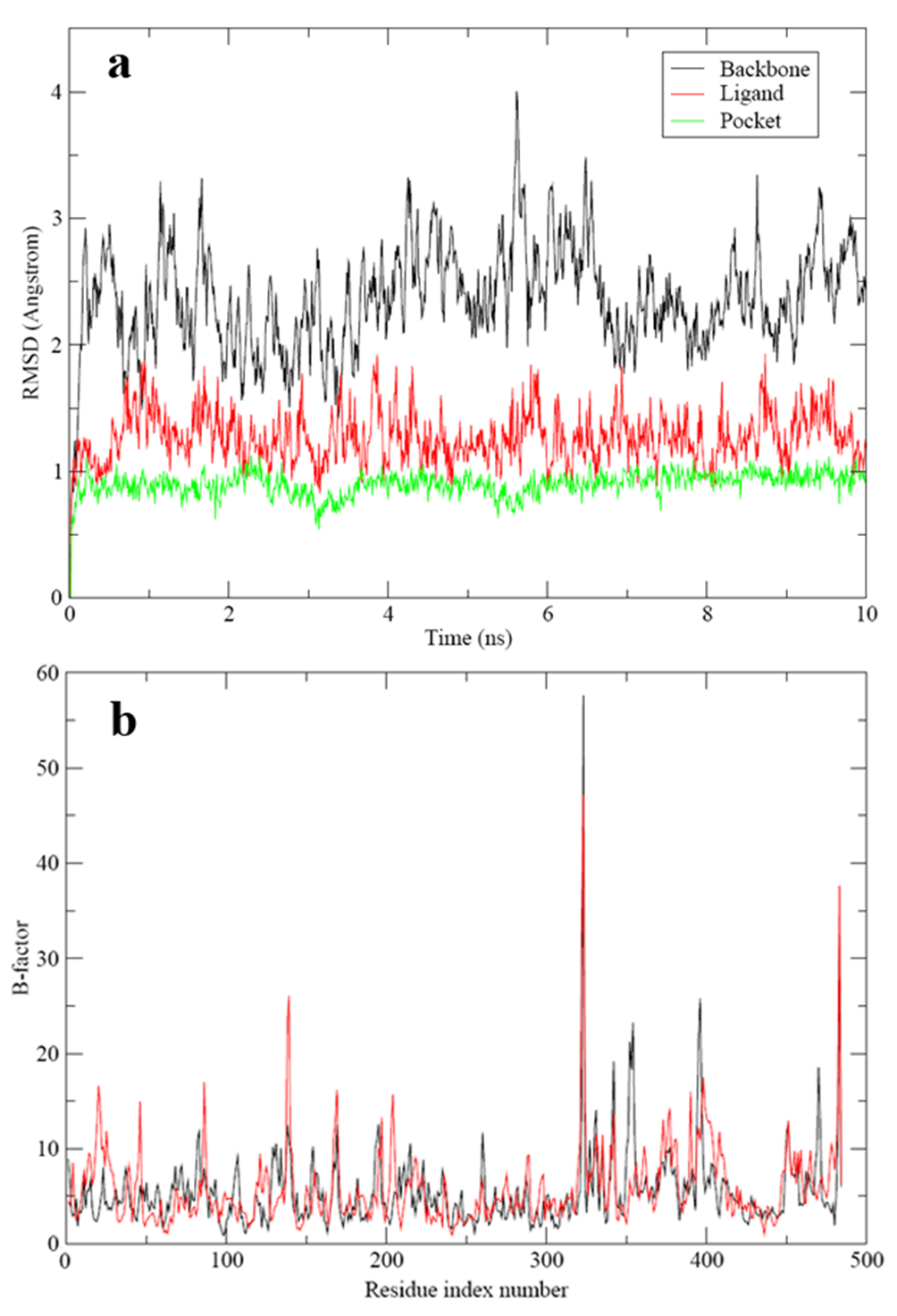

Supplement: Figure S2 — MD quality analyses by (a) RMSD values of receptor backbone, ligand and pocket residues are in black, red and green lines. (b) B-factor values of HA before/after MD are in black/red lines. Due to space limitation, only one system’s result is shown to be representative. (TIF) [file pone.0094392.s002.tif]

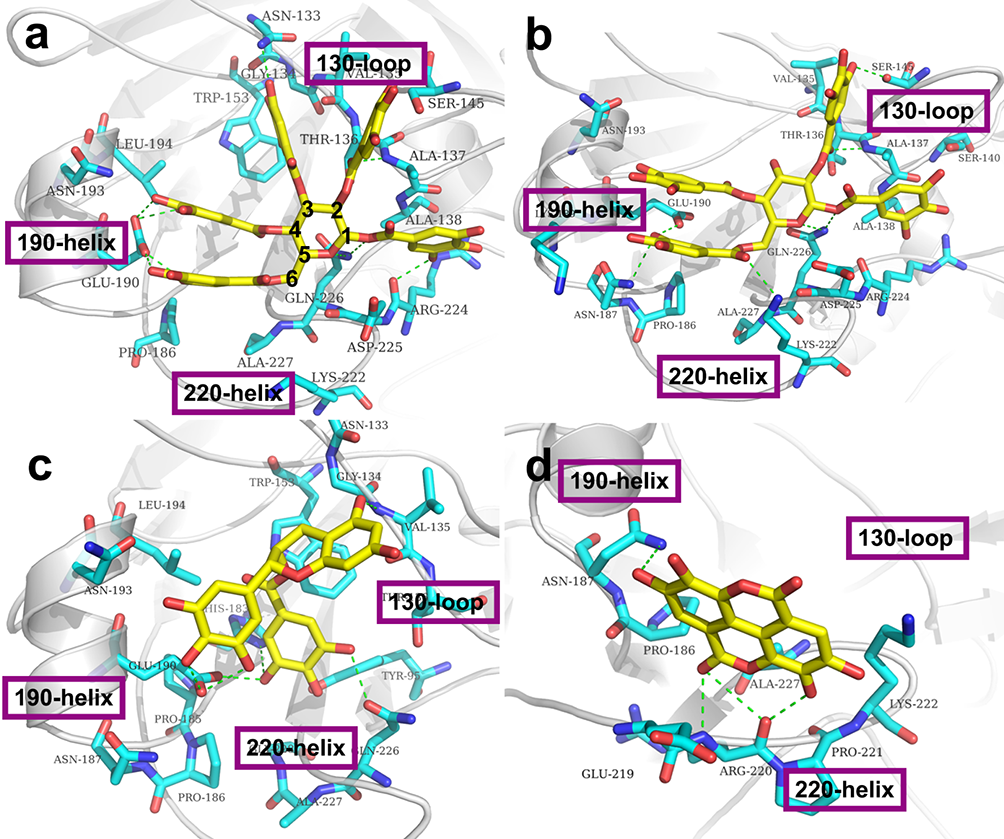

Supplement: Figure S3 — Representative binding modes of the most popular MD conformations from the trajectories of poly-galloyl-glucose analogs docked with HA/PR8. The secondary structure of the proteins is in grey, PGG and its analogues are colored in yellow: (a) PGG (b) TGG (c) EGCG (d) EA, pocket residues within 5 Å distance from the ligand are colored in cyan. Hydrogen bonds are depicted in green dash lines. (TIF) [file pone.0094392.s003.tif]

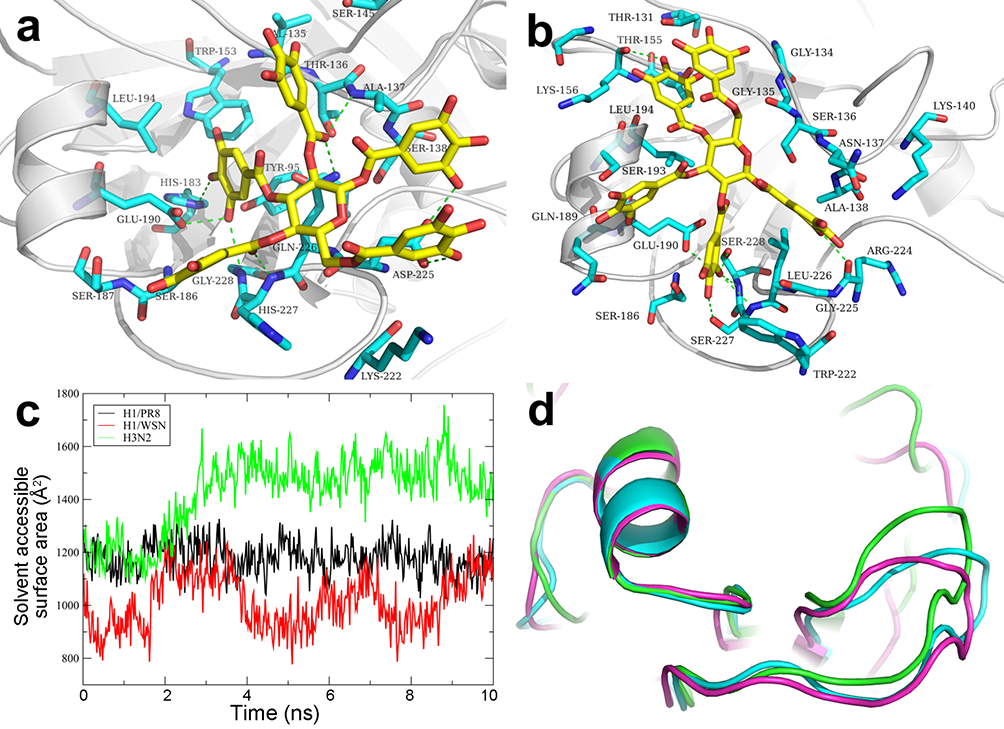

Supplement: Figure S4 — The most popular binding modes derived from MD simulations for PGG docked with (a) H1/WSN, and (b) H3/HK. The secondary structure of the proteins is in grey, PGG is in yellow sticks, receptor residues within 5 Å distance from the ligand are represented in cyan sticks. Hydrogen bonds are depicted in green dash lines. The three HA proteins, H1/PR8(cyan), H1/WSN(magenta), H3/HK(green) were superimposed to compare the secondary structure variations upon PGG binding. (c) Time series of solvent-accessible surface area (SASA) of H1/PR8, H1/WSN and H3N2 pocket residues upon PGG binding, (d) side view of the HA pocket after superimposing the three HA proteins. (TIF) [file pone.0094392.s004.tif]

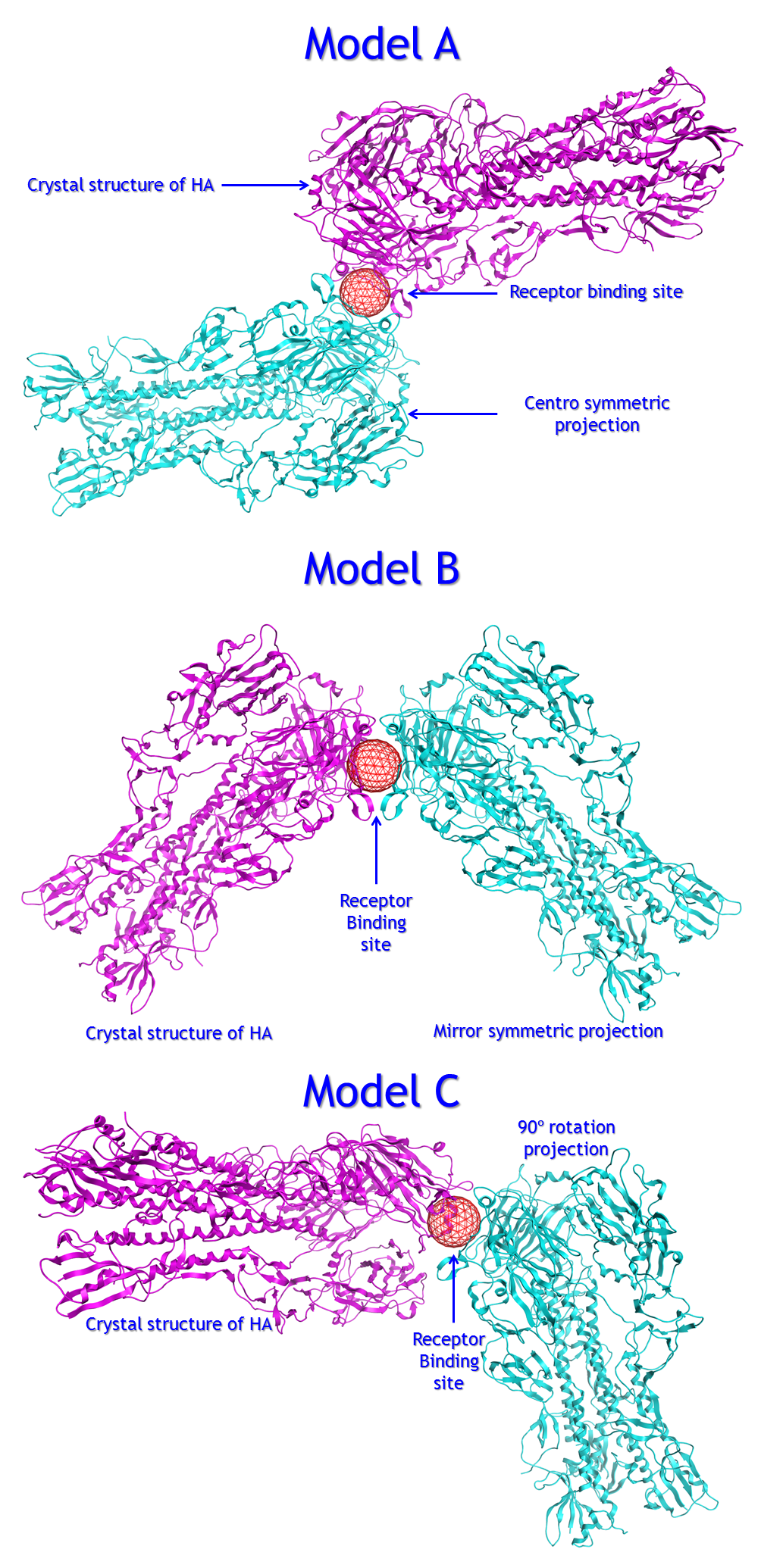

Supplement: Figure S5 — Three proposed models of PGG gluing hemagglutinin oligomers. HAs were represented by magenta and cyan ribbons. The red sphere marked the PGG binding site. The three models were built by creating the symmetric projection of the original HA and adding some rotation. (a) Trans-oriented model, created by centro symmetric projection (b) Cis-oriented model, created by mirror symmetric projection (c) Orthogonal-oriented model, created by 90° rotation of mirror symmetric projection model. (TIF) [file pone.0094392.s005.tif]

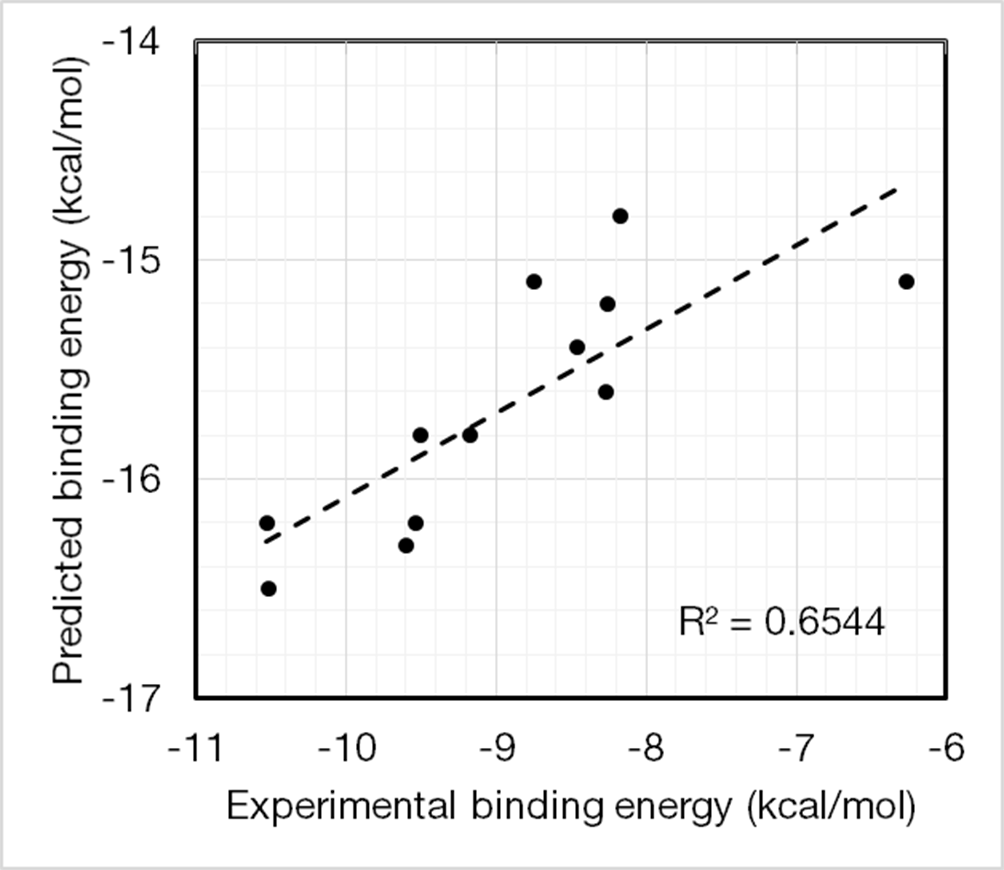

Supplement: Figure S6 — Correlation plot of predicted and experimental binding energy. The experimental binding energy is obtained by converting experimental KD values using formula ΔGbinding = RTlnK. (TIF) [file pone.0094392.s006.tif]

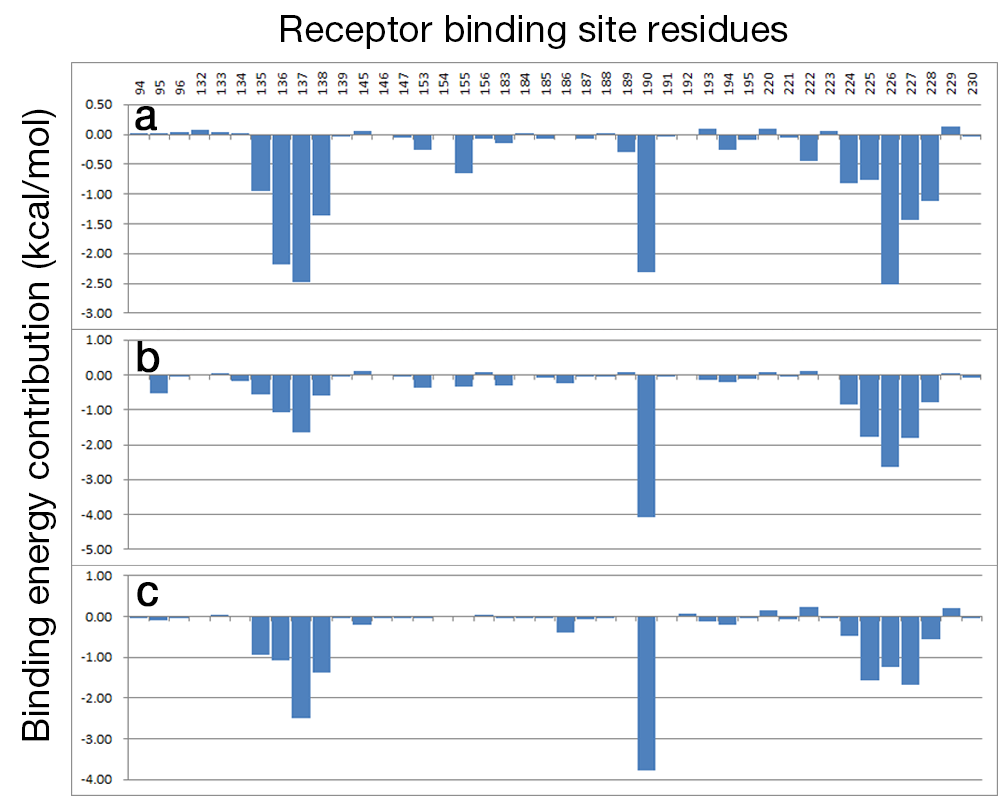

Supplement: Figure S7 — Binding energy decomposition results for another three systems. (a) PGG & H1N1/2009, (b) PGG & H5N1, (c) PGG & H7N9. (TIF) [file pone.0094392.s007.tif]
