# Supplementary material for: The Mechanism of Poly-Galloyl-Glucoses Preventing Influenza A Virus Entry into Host Cells
Source: PLoS One. 2014 Apr 9;9(4):e94392. doi: 10.1371/journal.pone.0094392 (PMC3981784; doi:10.1371/journal.pone.0094392)
Supplement: Table S1 — Sequence identity between the HA strains for bioassay and the available HA 3D structures. (DOC) [file pone.0094392.s008.doc]

**Table S1: Sequence identity between the HA strains for bioassay and the available HA 3D structures**

| **SeqA** | **Name** | **Length** | **SeqB** | **Name** | **Length** | **Score** |
| --- | --- | --- | --- | --- | --- | --- |
| **1** | A/HK/8/68_H3N2 | 566 | 2 | PDB:1RVZ | 487 | 42 |
| **1** | A/HK/8/68_H3N2 | 566 | 3 | A/WSN/33_H1N1 | 565 | 41 |
| **1** | A/HK/8/68_H3N2 | 566 | 4 | A/PR8/8/34_H1N1 | 565 | 40 |
| **1** | A/HK/8/68_H3N2 | 566 | 5 | PDB:5HMG | 503 | 99 |
| **2** | PDB:1RVZ | 487 | 3 | A/WSN/33_H1N1 | 565 | 89 |
| **2** | PDB:1RVZ | 487 | 4 | A/PR8/8/34_H1N1 | 565 | 98 |
| **2** | PDB:1RVZ | 487 | 5 | PDB:5HMG | 503 | 42 |
| **3** | A/WSN/33_H1N1 | 565 | 4 | A/PR8/8/34_H1N1 | 565 | 90 |
| **3** | A/WSN/33_H1N1 | 565 | 5 | PDB:5HMG | 503 | 43 |
| **4** | A/PR8/8/34_H1N1 | 565 | 5 | PDB:5HMG | 503 | 42 |
