# Supplementary material for: The Mechanism of Poly-Galloyl-Glucoses Preventing Influenza A Virus Entry into Host Cells
Source: PLoS One. 2014 Apr 9;9(4):e94392. doi: 10.1371/journal.pone.0094392 (PMC3981784; doi:10.1371/journal.pone.0094392)
Supplement: Table S2 — Binding affinity calculation results for each conformation and each model. (DOC) [file pone.0094392.s009.doc]

**Table S2** Binding affinity calculation results for each conformation and each model.

| **Conf.** | **Model A** | |  | **Model B** | |  | **Model C** | |
| --- | --- | --- | --- | --- | --- | --- | --- | --- |
|  | **ΔH** | **SEM** |  | **ΔH** | **SEM** |  | **ΔH** | **SEM** |
| **1** | -104.20 | 1.11 |  | -78.99 | 2.06 |  | -74.63 | 1.01 |
| **2** | -97.80 | 1.33 |  | -76.03 | 0.91 |  | -71.33 | 0.91 |
| **3** | -89.87 | 1.87 |  | -61.77 | 1.43 |  | -66.34 | 1.12 |
| **4** | -78.28 | 0.96 |  | -61.61 | 0.92 |  | -58.49 | 0.83 |
| **5** | -72.44 | 1.49 |  | -61.53 | 0.71 |  | -57.05 | 0.93 |
| **6** | -68.71 | 1.42 |  | -56.76 | 3.11 |  | -52.20 | 1.61 |
| **7** | -66.42 | 0.85 |  | -55.37 | 1.31 |  | -51.67 | 1.61 |
| **8** | -66.39 | 1.21 |  | -51.86 | 2.08 |  | -50.72 | 1.39 |
| **9** | -61.30 | 1.56 |  | -49.85 | 1.40 |  | -49.36 | 2.22 |
| **Min.** | -104.20 |  |  | -78.99 |  |  | -74.63 |  |
| **Max.** | -61.30 |  |  | -49.85 |  |  | -49.36 |  |
| **Avg.** | -78.38 |  |  | -61.53 |  |  | -59.09 |  |

All the units are in kcal/mol. SEM, standard error of mean. The lower the ΔH, the higher the binding affinity.
