# Supplementary material for: The Mechanism of Poly-Galloyl-Glucoses Preventing Influenza A Virus Entry into Host Cells
Source: PLoS One. 2014 Apr 9;9(4):e94392. doi: 10.1371/journal.pone.0094392 (PMC3981784; doi:10.1371/journal.pone.0094392)
Supplement: Table S3 — Various energy contributions to the binding energy for each complex system of pGG analogs vs. mono HA. (DOC) [file pone.0094392.s010.doc]

**Table S3** Various energy contributions to the binding energy for each complex system of pGG analogs vs. mono HA.

| Receptor | H1N1/PR8 | H1N1/WSN | H3N2 | H1N1/PR8 | H1N1/PR8 | H1N1/PR8 |
| --- | --- | --- | --- | --- | --- | --- |
| Ligand | PGG | PGG | PGG | TGG | EGCG | EA |
| ΔE_vdw_ | -38.82(1.22) | -42.97(1.43) | -44.93(0.76) | -32.50(1.09) | -18.91(1.06) | -14.16(0.91) |
| ΔE_ele_ | -93.43(2.69) | -79.25(2.41) | -33.68(1.97) | -81.95(2.42) | -81.24(3.16) | -46.79(3.70) |
| ΔE_ele,solv_ | 92.20(1.33) | 82.42(1.52) | 56.29(1.28) | 79.97(2.11) | 68.56(1.73) | 41.53(1.90) |
| ΔE_nonpol,solv_ | -4.14(0.06) | -3.89(0.06) | -4.21(0.04) | -3.16(0.05) | -2.51(0.02) | -1.13(0.04) |
| ΔG_gas_ | -132.25(2.96) | -122.23(2.81) | -78.61(2.11) | -114.45(2.65) | -100.15(3.33) | -60.95(3.13) |
| ΔG_solv_ | 88.06(1.33) | 78.53(1.52) | 52.08(1.28) | 76.81(2.11) | 66.04(1.73) | 40.40(1.88) |
| ΔH | -44.19(1.38) | -43.70(1.17) | -26.53(1.25) | -37.64(0.98) | -34.11(1.33) | -20.55(1.52) |
| -TΔS | 27.99(1.67) | 28.23(1.29) | 13.58(1.48) | 22.5(1.36) | 17.91(1.82) | 12.43(1.23) |
| ΔG_binding_ | -16.2(1.42) | -15.43(2.15) | -12.95(1.97) | -15.1(1.29) | -16.2(1.58) | -8.12(0.83) |

All the units are in kcal/mol. ^a^ Nonbonded van der Waals. ^b^ Nonbonded electrostatics.

^c^ Electrostatic component to solvation. ^d^ Nonpolar component to solvation.
