# Supplementary material for: The Mechanism of Poly-Galloyl-Glucoses Preventing Influenza A Virus Entry into Host Cells
Source: PLoS One. 2014 Apr 9;9(4):e94392. doi: 10.1371/journal.pone.0094392 (PMC3981784; doi:10.1371/journal.pone.0094392)
Supplement: Text S1 — Detailed methods for virus purification, redocking evaluation, BLAST, and binding energy calculations. (DOC) [file pone.0094392.s011.doc]

## Virus purification

Virus was purified using a sucrose density gradient centrifugation process. Briefly, allantoic fluid of infected chick embryos was collected and subjected topreliminary purification by centrifugation at 7,000×g (Hettich-Zentrifugen, Germany) for 30 minutes at 4°C. The supernatant was gathered for another 2 hours of centrifugation at 30,000×g (Beckman Coulter, USA) at 4°C and the obtained precipitate was suspended in pH7.2 PBS at 4°C overnight. The virus suspension was further loaded on 30-60% discontinuous sucrose density gradient prepared by 2mL of 60%, 4 mL of 45% and 4 mL of 30% sucrose solution for centrifugation at 100,000×g (Beckman Coulter, USA) for 2 hours at 4°C. Virus containing bands between 30% and 45% sucrose solution were collected, diluted in pH7.2 PBS and subjected to further centrifugation at 100,000×g (Beckman Coulter, USA) for 90 minutes at 4°C to remove sucrose. The purified viruses were then suspended in pH7.2 PBS and stored in liquid nitrogen if not used immediately.

## Evaluation of docking programs by re-docking experiments

The 5 docking programs, MOE 2010, CDOCKER of Discovery Studio (DS) 2.5, Ligandfit of DS 2.5, Surflex of Sybyl 7.3 and FlexX of LeadIT 1.0 were used to dock the original ligand of the PDB 1RVZ back into the ligand binding site. RMSD values of the poses before/after docking were calculated and plotted as **Fig. S1**.

## BLAST analysis

The HA sequences of the H1N1 and H3N2 influenza virus strains adopted in this study were downloaded from the PubMed website. BLAST analysis was carried out on the PDB database and located PDB 1RVZ and 5HMG. H1/PR8 and H1/WSN showed high homology with PDB 1RVZ. H3/HK8 showed high homology with PDB 5HMG. The sequence identity matrix was calculated by MOE and listed in **Table S1**.

According to BLAST, it would be safe to adopt the two PDBs for simulating H1N1 and H3N2 HAs without additional homology modelling procedure. Only the residues around 8 Å of the receptor binding site of HA were modified on the crystal structures according to the actual bioassay HA sequences to grant consistency between *in vitro* and *in silico* experiments.

## Quality analyses of MD simulations

The root-mean-square deviation (RMSD) and b-factor values of the simulated systems were calculated using the Ptraj program of AMBER Tools v1.5. A representative set of RMSD and b-factor values were plotted as **Fig. S2**. As can be seen, the system reached equilibrium within 10 ns MD simulation and the pocket residues were quite stable.

## Binding energy calculations

Binding energy was calculated to obtain information on ligands bound HA from energetic aspects. The free energy of binding, ΔG_binding_, was calculated according to Eq. (1) from the free energy of the receptor-ligand complex (G_cpx_) with respect to the unbound receptor (G_rec_) and ligand (G_lig_):

ΔG_binding =_ G_cpx_ – (G_rec_ + G_lig_) (1)

The MM-PBSA (Molecular Mechanics-Possion-Boltzmann/Surface Area) methodology allows the calculation of the complete binding reaction energy, including the desolvation of the ligand and the unbound protein, on the basis of a thermodynamic cycle. Therefore, Eq. (1) can be approximated as

ΔG_binding =_ ΔE_MM_ – TΔS + ΔG_sol_ (2)

ΔE_MM =_ ΔE^ele^ + ΔE^vdw^ (3)

All energies represented in the above equations were averaged over the course of the molecular dynamics trajectories. In Eq. (3), ΔE_MM_ is the molecular mechanical energy obtained from the electrostatic (ΔE^ele^) and the van der waals (ΔE^vdw^) interactions within the system. Here, TΔS is the solute entropic contribution at temperature T (kelvin) and the solvation free energy (ΔG_sol_) represents the electrostatic and nonpolar free energy of solvation, and therefore can be expressed as


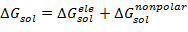
 (4)

where
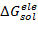
 is the polar contribution to solvation and
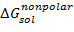
 is the nonpolar solvation term. The former component was calculated using the PB calculation, whereas the latter term is determined using Eq. (5):


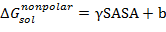
 (5)

Where SASA is the solvent-accessible surface area (Å^2^) and
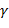
 and *b* represent experimental solvation parameters. The ΔG_binding_ value was obtained using the MM-PBSA module in the program AMBER 11.
